# Predicting stage-specific cancer related genes and their dynamic modules by integrating multiple datasets

**DOI:** 10.1186/s12859-019-2740-6

**Published:** 2019-05-01

**Authors:** Chaima Aouiche, Bolin Chen, Xuequn Shang

**Affiliations:** 1 School of Computer Science, Northwestern Polytechnical University, Xi’an, 710072 China; 2 Key Laboratory of Big Data Storage and Management, Northwestern Polytechnical University Ministry of Industry and Information Technology, Xi’an, China

**Keywords:** Disease genes, Clinical stages, Dynamic modules, Pathway networks, Disease evolution

## Abstract

**Background:**

The mechanism of many complex diseases has not been detected accurately in terms of their stage evolution. Previous studies mainly focus on the identification of associations between genes and individual diseases, but less is known about their associations with specific disease stages. Exploring biological modules through different disease stages could provide valuable knowledge to genomic and clinical research.

**Results:**

In this study, we proposed a powerful and versatile framework to identify stage-specific cancer related genes and their dynamic modules by integrating multiple datasets. The discovered modules and their specific-signature genes were significantly enriched in many relevant known pathways. To further illustrate the dynamic evolution of these clinical-stages, a pathway network was built by taking individual pathways as vertices and the overlapping relationship between their annotated genes as edges.

**Conclusions:**

The identified pathway network not only help us to understand the functional evolution of complex diseases, but also useful for clinical management to select the optimum treatment regimens and the appropriate drugs for patients.

## Introduction

Complex diseases, such as cancers, are kinds of evolutionary diseases [1, 2], which involve successive stages from early initiation to advanced end-stages. Determining the possible biological changes associated with these stages is necessary for understanding the progression of many diseases, thereby specifying their best treatment strategy. Take the colorectal cancer for example, these stages can be classified generally into four phases based on their level of extension, lymphatic involvement and metastatic features. Specifically, stage I refers to a tumor of small size confined to the organ of origin; stage II describes the disease that has locally advanced beyond the site of origin; stage III characterizes the disease that has spread to the neighboring organs; and stage IV represents distant metastatic disease. Here, cancers at early stages (stage I or II) are usually considered curable and might only need an active surveillance compared to advanced stages (stage III or IV) which might require more radical and active treatment. Therefore, the understanding of the biological mechanism and molecular events of complex diseases through stages require the identification of stage-specific disease genes, unlike other irrelevant genes and genetic aberrations that turned out to have no functional relevance to any disease or specifically to cancer biology [3].

The availability of high dimensional datasets, and the great advancement of high-throughput technologies have enabled the identification of genes associated with specific diseases, providing potential methods for precision medicine [4] and drug design [5]. Take the Cancer Genome Atlas (TCGA) project for example, it has generated multi-omics datasets over the genomic, epigenetic and transcriptome levels together with clinical data for more than 30 human tumors [6–9]. These multiple omics datasets provided many high-resolution molecular profiles, such as gene expression (microarray, RNA-seq), copy number variation (CNV or sCNA), DNA methylation, mRNA expression, somatic mutation, protein expression, as well as clinical information describing specific metrics, which including pathological stages, clinical stages, grade and age at diagnosis. They are highly variable in term of availability from disease to disease.

These datasets enabled integrative analysis focusing on the identification of cancer-related genes [10–14], unlike individual analysis with a single type of data, which represents an incomplete snapshot of a biological process and does not provide a comprehensive view of different disease states. In addition, clinical data also provided valuable insights into the genetic aberration detections, including cancer genes identification and their clinical translation [15–17].

Despite many discoveries made by these integrated genome datasets, there are only a limited number of studies that consider the associations between genomic profiles, clinical parameters, and their stage related cancer genes [18–24]. Moreover, these discoveries often neglected the fact that those identified cancer genes and functional modules are dynamically changed. Identifying the evolution of these biological modules is very important to understand the progression of many complex diseases, the key regulators of many cancer-related genes and their dysregulated pathways [25–31].

The main objective of this paper is to investigate a versatile working flow that can address the staging evolution processes of complex diseases, which including: (1) the identification of stage-specific cancer related genes, (2) the construction of their related dynamic modules, and (3) the generation of the stage related pathway networks. The rest of the paper is organized as follows. “Materials and methods” section introduces the methods and related materials. “Results and discussions” section addresses the numerical experiments and results. “Conclusion” section draws discussions and conclusions.

## Materials and methods

### Data sources and preprocessing

The Level 3 clinical information and genomic datasets were obtained from the FIREHOSE Broad GDAC [32]. It is one of the Genome Data Analysis Centers (GDACs) for the TCGA project that are used for prognosis and disease diagnosis. The datasets were downloaded in December 2016, which including the clinical information, gene expression and DNA methylation profiles for the same group of patients. The summarized information can be found in Table 1.

**Table 1:** The datasets informations for the same set of samples from Broad Firehose TCGA project

Data type	Platform	Samples
Gene expression	UNC-AgilentG4502A	219
DNA methylation	JHU-USC-HumanMethylation27	219
Clinical data	-	219

The clinical information for each patient are highly variable. Therefore, we focus on the “pathology_t_stage”, which mainly describes the diagnosis stage of individual samples (*t*_1_,*t*_2_,*t*_3_ and *t*_4_). These pathological variables were converted into binary values for our following regularized regression analysis. For the sample selection, we only take those patients when the “pathology_t_stage” parameter was available. Finally, 219 samples were used to conduct our subsequent analysis.

The gene expression and DNA methylation profiles were measured for majority genes, which contain 17505 gene expressions and 26224 DNA methylations. However, we only consider the intersection of the two gene sets, which contain both gene expression and DNA methylation information in datasets. Moreover, genes with missing values, such as NA or NULL, were filtered out, and methylation CpG loci which related to multiple genes were shared equally for those genes. Eventually, a set of 12586 genes were obtained in this study.

**Fig. 1:**
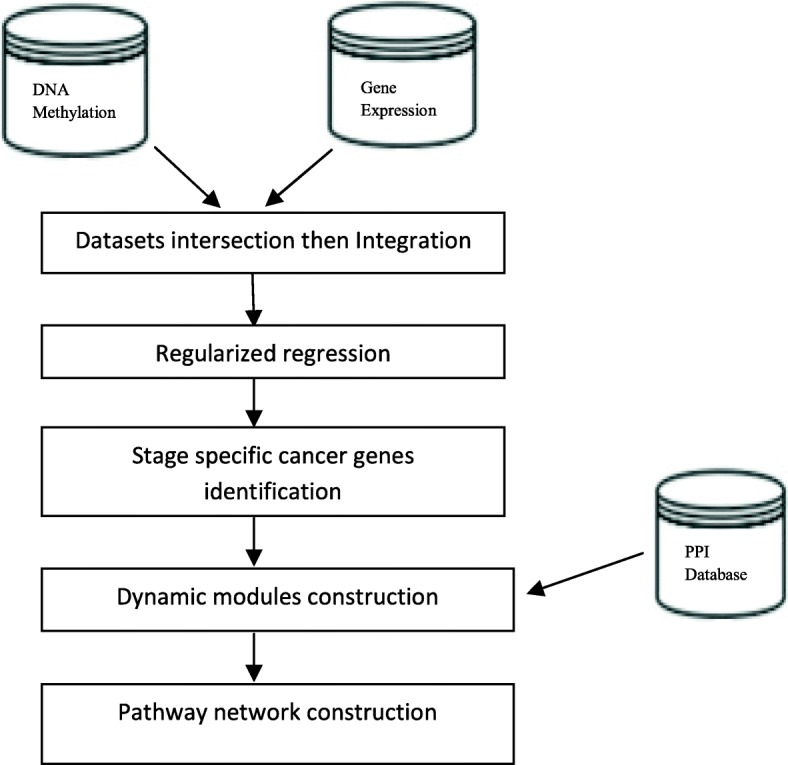
The workflow for identifying stage-specific cancer related genes and their evolution processes through pathological_staging

Additionally, the HPRD PPI network (release 9) [33] were used to detect gene interactions and functional modules, which contained 9465 proteins and 37039 interactions. The workflow of the whole process is shown in Fig. 1.

### Stage-specific related gene identification

The first important step to investigate the evolution progress of complex diseases is to identify signature genes for individual stages. Elastic net [34] is one of the classical feature selection algorithms, and has been widely used in biological and clinical research areas [35–42]. It employs a generalized linear regression model to handle the high-dimensional data regression issue without using any prior information. The elastic net method is based on a compromise between the Least Absolute Shrinkage and Selection Operator (LASSO) penalty (L1 norm) and the ridge penalty (L2 norm), where the LASSO penalty performs the feature selection and the coefficient estimation, while the ridge penalty shrinks those coefficients toward to zero [35].

Suppose there is a set of *m* samples (patients) and *n* features (gene expression or methylation profiles), the feature matrix can be denoted as a *m*×*n* dimension matrix *X*. Given a *m* dimension label vector *Y* (pathology stage labels), the problem stage-specific related gene identification is to detect a set of genes that minimize the following objective function

1 $$\begin{array}{@{}rcl@{}} B = \| Y-X\beta \|_{2} \end{array} $$

where *β*=(*β*_1_,*β*_2_,…,*β*_*n*_)^*T*^ is the coefficient vector for all features.

After adding a LASSO penalty and a ridge penalty, the elastic net method have a form like

2 $$\begin{array}{@{}rcl@{}} \widehat B = \| Y-X\beta \|_{2} + \lambda_{1} | \beta | + \lambda_{2} \| \beta \|_{2}, \end{array} $$

or

3 $$ {\begin{aligned}  \widehat B = argmin \left\lbrace \frac{1}{m} \sum\limits_{{i}=1}^{m}{\left({{y}}_{{i}}- \sum\limits_{j=1}^{n}{{{x}}_{{ij}}}\beta_{j} \right)}^{2} +\lambda_{1} \sum\limits_{{j}=1}^{n}\ {|\beta_{{j}}}|+ \lambda_{2} \sum\limits_{{j}=1}^{n} {\beta_{{j}}}^{2} \right\rbrace \end{aligned}}  $$

to be more specific, where *λ*_1_,*λ*_2_ are the penalty parameters related to LASSO and ridge penalty, respectively.

In this study, the gene expression profiles and the DNA methylation information were integrated to form the feature matrix *X*, and four binary stage-specific label vectors *Y*_*t*_,*t*=1,2,3,4 were employed to identify disease related genes for individual stages, respectively (where an element in *Y*_*t*_ represents if that sample was recognized as the *t*_*th*_ pathology stage in the clinical dataset).

The objective function (3) was implemented in Matlab R2015a with the tuning parameter *λ*_1_=*λ*_2_=0.5. The fitted least-squares regression coefficients were used for gene selection. Giving a pair of *X* and *Y*_*t*_, the Matlab program calculated the fitted coefficients at around 50 times (automatically determined by Matlab). At each time, a set of signature genes could be selected if their coefficients were larger than a threshold. The finally stage-specific genes were determined based on the times of those genes were selected during the calculation. Table 2 summarizes the times of running, the number of selected genes across the 4 pathology stages, and the number of genes that were selected at least 20 times.

**Table 2:** The number of genes detected by cut off=20

Stages	Models #	Detected # of genes	# of genes at cut off=20	# of genes in the giant components
Pathology_t1	51	279	167	17
Pathology_t2	48	257	195	40
Pathology_t3	45	272	206	227
Pathology_t4	50	278	178	64

[20 implies the number of genes selected by at least 20 models]. This table illustrated the number of the models resulted at each pathology_stage, the maximum number of non-zero coefficients (genes) obtained at a specific model, the number of genes predicted by a cutoff metric and the number of genes in the giant components

### Stage-specific module detection

Stage-specific modules at each pathology stage were constructed based on the giant component strategy and the human PPI network.

The selected signature genes at each stage were often isolated with each in many cellular networks, and the enrichment analyses of those genes may not get any meaningful result. To overcome this problem, we propose to use the giant component strategy to select the most functional related gene modules based on the identified genes.

**Fig. 2:**
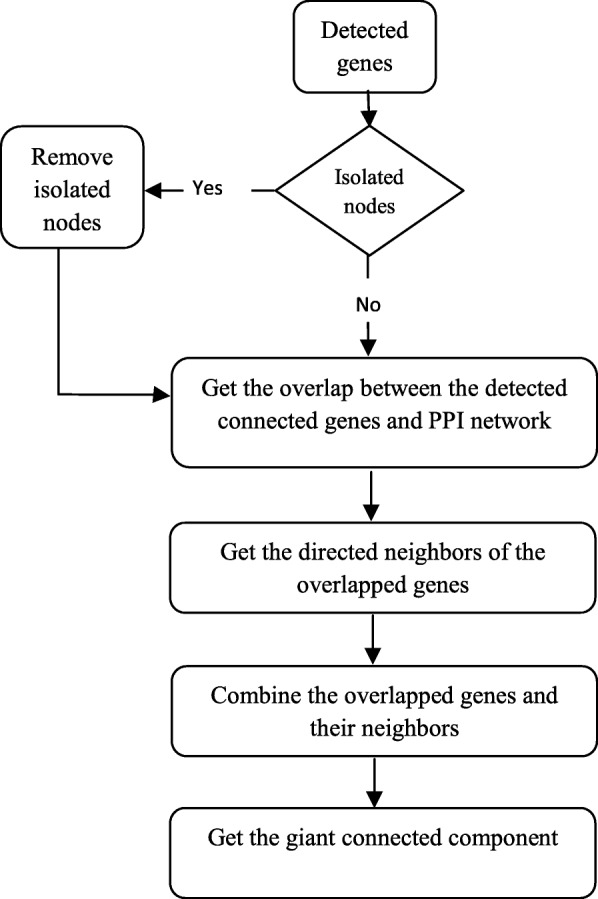
The flow chart of the stage-specific gene identification

To be more specific, a biological network could be employed as the basic background network, where the identified genes and their directed neighbors in the network were selected to form a subnetwork. The edges of the subnetwork were also generated based on the background network. By doing this, the obtained subnetwork often robustly linked with each other, and the initial identified genes served as seed nodes to generate the related functional modules. To further filtering out those disrelated genes, only the genes belong to the giant connected component of the subnetwork were selected as the signature genes for that stage. The rest of other genes will not consider in this study. The flowchart of this part was illustrated in Fig. 2.

The HPRD PPI dataset deemed the most important interaction in this study compared to other human datasets like the human cofunction network in [43] and the InWeb_IM PPI network in [44], since it linked efficiently the genes identified at each stage.

### Dynamic module analysis and pathway network generation

Once the signature genes were identified for each stage, the Cytoscape was used to draw the explicit graphical representations for different biological modules and subnetworks. In this study, 4 groups of dynamic giant connected functional modules were constructed, where the vertices represented the list of interested genes at each stage, and edges represented the functional relations between them obtained from the PPI network.

To further determine whether the list of signature genes identified at individual pathology stages are statistically enriched in certain biological processes or functions, functional enrichment analysis were performed using the DAVID tool [45]. A list of significant Reactome pathways has been obtained from these enrichment analysis. We then pooled these Reactome pathways altogether and get their official annotated pathway descriptions from the database. Next, a pathway evolution network was generated by pooling all those stage-specific pathways together, where the vertices in this network represent individual pathways, and connections were obtained if the two pathways have overlapped genes.

The pathway network could clearly show the dynamic evolution processes of the interested disease, since we can use the color of individual vertices to indicate their pathology stage, and the width of edges can show the overlapped score between two pathways. Here, the overlap score was calculated as follows:

4 $$\begin{array}{@{}rcl@{}} W= \frac{k^{2}}{p*q}. \end{array} $$

where *k* is the number of the overlapped genes between a pair of pathway *P*_*i*_ and pathway *P*_*j*_, *p* and *q* are the total numbers of genes in *P*_*i*_ and *P*_*j*_, respectively.

## Results and discussions

### The number of stage-specific related genes

In this study, we have selected those genes that were detected by at least 20 models as the seed of stage specific related genes. By using this strategy, a list of signature genes that robustly delineate early and advanced pathological stages. Table 2 summarized the number of genes selected at different stages. To be more specific, stage t1 has obtained 167 genes from 51 models; stage t2 has obtained 195 genes from 48 models; stage t3 has obtained 206 genes from 45 models; and stage t4 has obtained 178 genes from 50 models, respectively.

All of these genes were considered as indicators or signatures to characterize the dynamics of the 4 pathological stages, due to their possible role in cancer progression.

### Dynamic modules construction and visualization

The HPRD network was used to construct 4 groups of pathology stage related modules based on their identified giant components. Interactions among their identified genes were extracted to form the corresponding modules, which contained 17 nodes and 23 interactions for stage t1; 42 nodes and 51 interactions for stage t2; 228 nodes and 1004 interactions for stage t3; and 65 nodes and 87 interactions for stage t4.

**Fig. 3:**
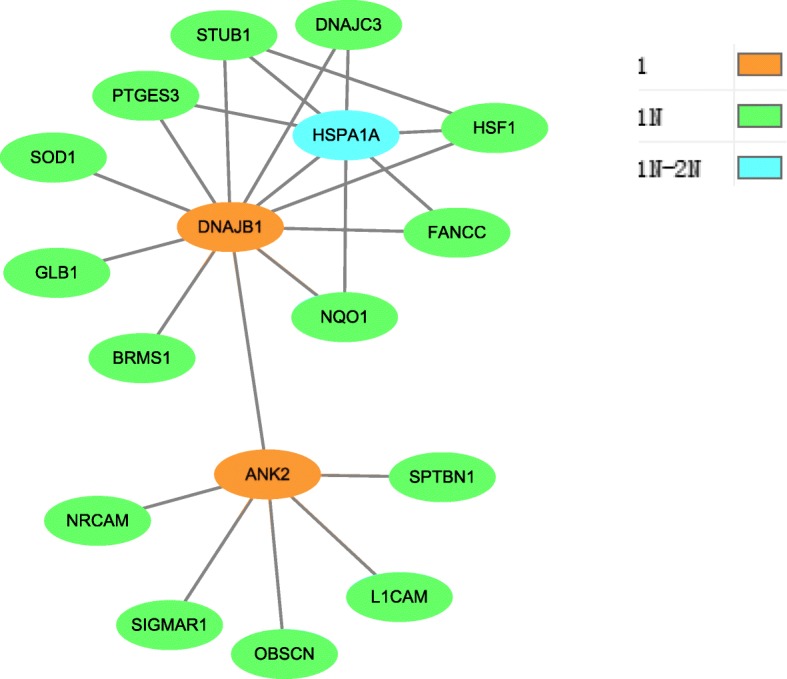
Pathology_t1 stage module. This module has 17 giant component nodes (genes) interacted with 23 edges. Node colors specify: stage1 identified genes, their neighbors and also the overlapped genes from other pathology stages, where 1 indicates stage1 detected genes, 1N indicates stage1 directed neighbors and 1N-2N indicates the overlapping genes between stage1 neighbor genes and stage2 neighbor genes as shown in the code colors

**Fig. 4:**
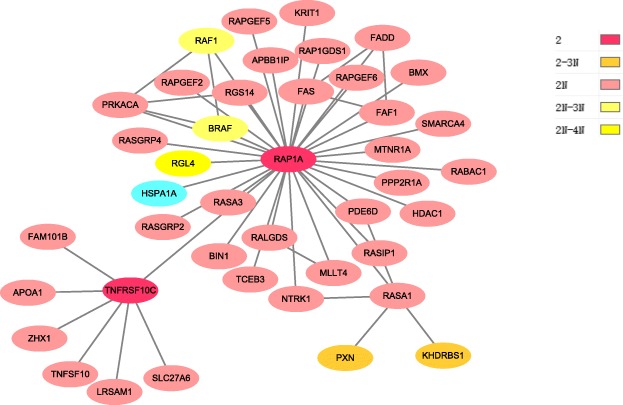
Pathology_t2 stage module. This module has 42 giant component nodes interacted with 51 edges. Node colors specify: stage2 identified genes, their neighbor genes and also the overlapped genes from other pathology stages, where 2 indicates stage2 detected genes, 2N indicates stage2 directed neighbors, 2-3N indicates the overlapping genes between stage2 detected genes and stage3 neighbor genes, 2N-3N indicates overlap genes between stage2 neighbor genes and stage3 neighbor genes and 2N-4N denotes overlap genes between stage2 neighbor genes and stage4 neighbor genes which shown clearly in the code colors

**Fig. 5:**
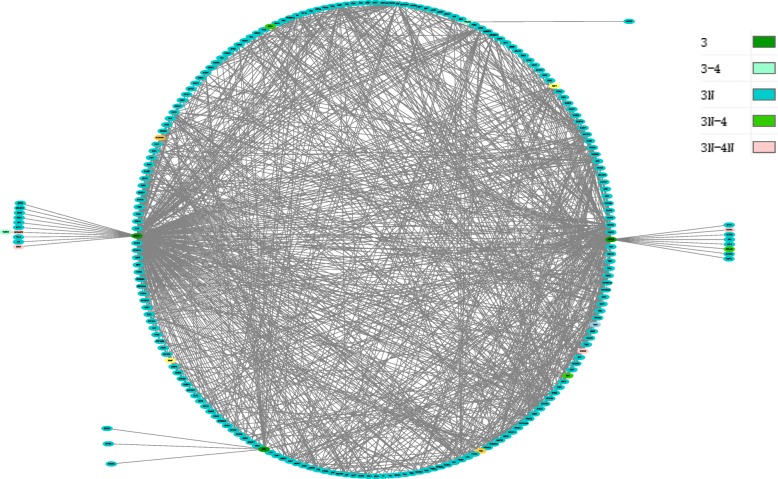
Pathology_t3 stage module. This module has 228 giant component nodes interacted with 1004 edges. Node colors specify: stage3 identified genes, their neighbor genes and also the overlapped genes from other pathology stages, where 3 indicates stage3 detected genes, 3N indicates stage3 directed neighbors, 3-4 indicates the overlapping genes between stage3 detected genes and stage4 detected genes, 3N-4 indicates overlap genes between stage3 neighbor genes and stage4 detected genes and 3N-4N denotes overlap genes between stage3 neighbor genes and stage4 neighbor genes which shown clearly in the code colors

**Fig. 6:**
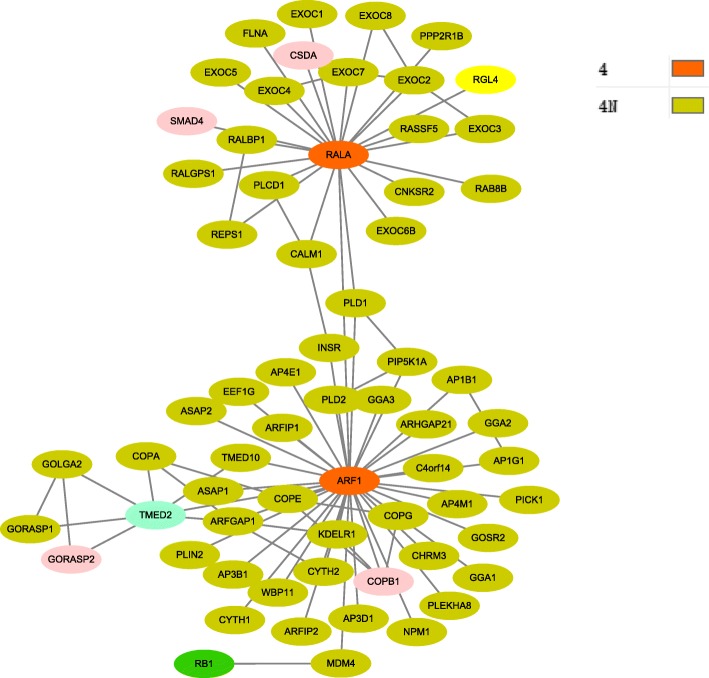
Pathology_t4 stage module. This module has 65 giant component nodes interacted with 87 edges. Node colors specify: stage4 identified genes, their neighbor genes and also the overlapped genes from other pathology stages, where 4 indicates stage4 detected genes, 4N indicates stage4 directed neighbors. In addition to the overlapped genes from stage 2 and 3 as shown in previous code colors

In order to further know how the four pathology stages involved and interacted to each other, the overlapping cancer genes between them were identified from the combined set, and the connections of these genes along with their neighbors at individual stage compared to other stages were shown in Figs. 3, 4, 5 and 6, respectively. These figures show originally detected genes, neighbor genes and their overlapped genes of individual pathology stages, which are highlighted by different colors.

### Annotated functions and pathway enrichment analysis

Pathway analysis has become the first choice for gaining insight into the underlying biology of differentially expressed and methylated genes, as it reduces complexity and has increased explanatory power. Thus, to validate our results, a DAVID functional annotation tool with a Reactome pathway annotation [46] was carried out for the identified pathological significant genes to identify their potential pathways and thereafter construct the corresponding pathway network.

In this study, a considerable number of stage-specific genes have been successfully identified across 4 pathological stages. These identified genes robustly associated with each other to produce meaningful biological modules, and significantly enriched in some key biological pathways. Those pathways, in turn, also interacted at a higher level based on the overlapping between their annotated genes. The rich set of interactions between these pathways results in a valuable pathway network, capturing highly evolved pathways through the 4 pathological stages.

**Fig. 7:**
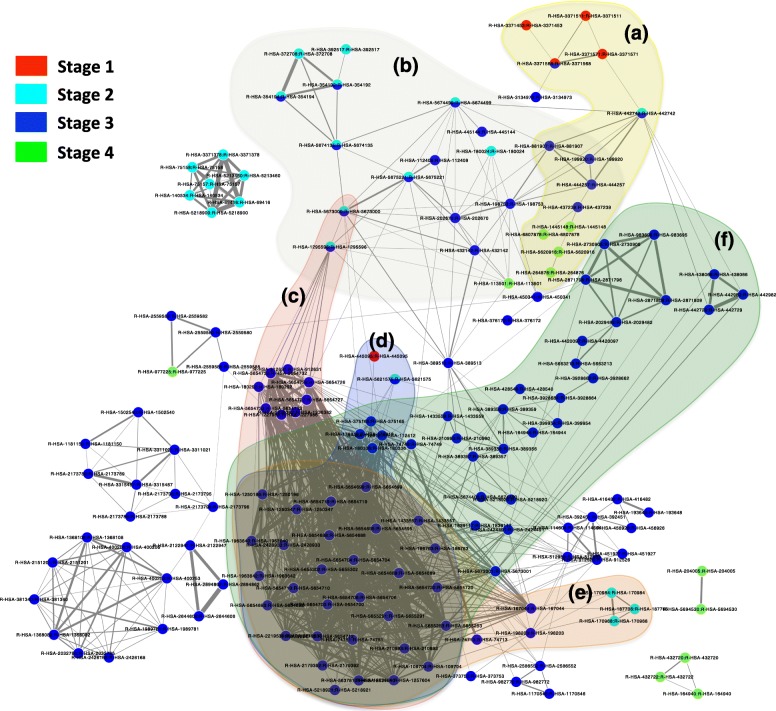
The dynamic structure of the pathway interaction network. The nodes in this network referred to pathways and edges between nodes indicated interaction of pathways determined through overlapped genes. In particular, nodes in red indicate stage1 pathways; nodes in blue indicate stage2 pathways; nodes in dark blue indicate stage3 pathways ;nodes in green indicate stage4 pathways, whereas nodes in 2 colors refer to the common pathways of 2 stages. The color of nodes represent the cancer stages

The network provides novel insights into the cancer disease evolution. As can be seen in Fig. 7, the evolution histories or communities could be clearly classified into 6 groups: (a), (b), (c), (d), (e) and (f). Specifically, in group (a), the cancer evolved from stage 1 (red nodes) to stage 2 (light blue nodes), and continued to evolve through stage 3 (dark blue nodes) to the end of stage 4 (green nodes). Similar to group (b) which involved an evolution start from stage 2 (blue node) to stage 2 and 3 by their common pathways (light blue and dark blue). Then, from stage 3 (dark blue) to stage 4 (which shown in green nodes). For group (c), an evolution also happened from stage 2 to stage 3 pathways. In group (d) the evolution happened through stage 1, stage 2 to stage 3, and in group (e) the evolution involved in stage 2 and stage 3. The final group (f), which includes a large set of stage 3 pathways strongly related to each other, suggesting the metastasis growth of cancer disease.

**Fig. 8:**
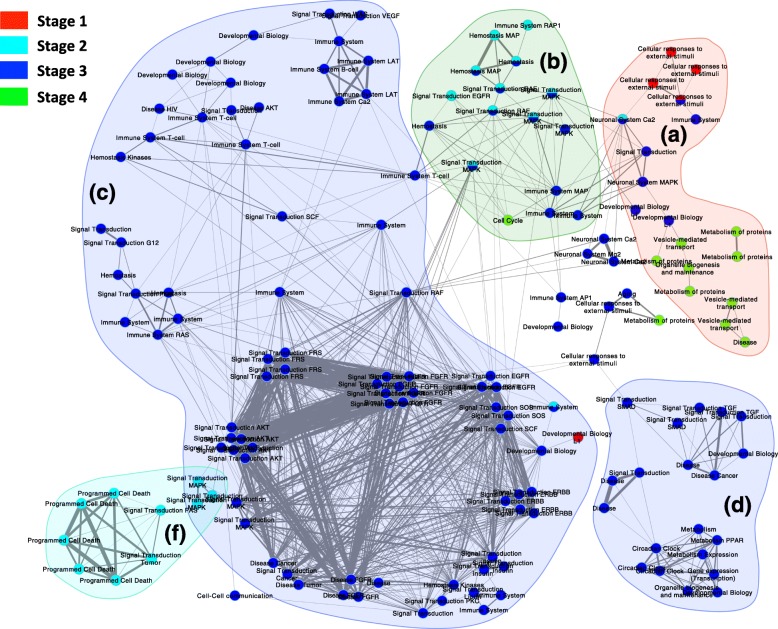
The annotated functions of the extracted pathway network. This figure illustrated the different pathways functions enriched at every pathology stage and the highlighted (**a**), (**b**), (**c**), (**d**) and (**f**) cases determine the main histories functions detected in this pathway network. The color of nodes represent the cancer stages

Moreover, to illuminate the biological significance role of the extracted pathway network and their evolved pathways, we also defined their annotated functions, which are shown in Fig. 8. For stage 1, the annotated functions mainly belong to cellular responses to external stimuli group. For stage 2, the annotated functions carry (1) immune system, (2) signal transduction tumor and (3) programmed cell death. For stage 3, the annotated functions evolve to (1) neuronal system, (2) immune system, (3) signal transduction and (4) developmental biology. The last stage includes annotated functions such as (1) metabolism of proteins, (2) vesicle mediated transport, (3) disease and (4) cell cycle.

To be more specific, for example, in the group (a), the evolved pathways were highly related to functions which starts from (1) cellular responses to (2) external stimuli, then go through (3) neuronal system signal transduction, (4) developmental biology, and ends up with (5) metabolism of proteins. In group (b), the evolution starts from (1) hemostasis to (2) signal transduction and ended with (3) cell cycle. Whereas in group (c), a set of communities enriched in common functions including (1) cancer, (2) disease and cancer, (3) disease and tumor, (4) signal transduction and (5) immune system. For group (f), the most pathways successfully enriched in programmed cell death function. However, in the group (d), different functions have been defined including (1) circadian clock and (2) gene expression (transcription). Overall, the description details of these functions suggesting the important biological role of this study.

## Conclusion

In this paper, we introduced a working flow which mainly adressed 3 important biological aspects such as stage-specific cancer genes identification, multi-omics data integration, biological modules and cellular pathway network construction.

The main objective of the study was to gain biological and clinical insights into the progression of cancer diseases through pathological staging mechanism. Since complex diseases, include but not limited to cancers, are evolutionary diseases that don’t directly end up with a mortal situation. They evolve cross multiple stages that can be determined not only through the lens of biological modules but more importantly through pathway networks, which contain rich biological information and provide more detailed molecular mechanisms.

Therefore, we constructed individual pathological modules based on the overlap between identified giant specific genes associated through a PPI network. We also performed pathway analysis on these genes and built a valuable pathway network based on the enriched pathways and the overlap between their annotated genes, which captured highly evolved pathways that involved different successive stages determining the real evolution of cancer diseases.

This process has furthered this understanding by identifying significant differences between different diseases stages and determining their evolution through pathways perspective, which have important implications not only for the classification of diseases/phenotypes, but also with clinical management by helping to select the most appropriate treatment modality for patients, holding promise for finding potential drugs.

Our understanding of cancer biology through the lens of the pathway and network analyses is promising. Especially when a disease reaches a metastasis status, which is the pivotal cause of patient deaths. The metastatic status is an advanced status that can be deeply defined by the TNM (Primary tumor (T), Lymph nodes (N) and Distant metastasis (M)) criteria, which are major parameters in the staging technique. Thus, we see ample opportunities to address this issue in future work. Furthermore, integrating more datasets at various levels (e.g gene expression, DNA methylation, and somatic CNV) might further facilitate the discovery of more robust staging modules and pathways, that easily determine the evolutionary process of many diseases revealing more comprehensive information of disease states. However, substantial additional experiments will be required to validate the predicted findings.

## Abbreviations

CNV: Copy number variation
DAVID: The database for annotation, visualization and integrated discovery
GDACs: Genome data analysis centers
HPRD: Human protein reference database
InWeb_IM: InWeb_InBioMap
LASSO: Least absolute shrinkage and selection operator
PPI: Protein-protein interaction
sCNA: Somatic copy number alteration
TCGA: The cancer genome atlas
TNM: Primary tumor, lymph node and distant metastasis
